# An Electrical Characterisation Methodology for Benchmarking Memristive Device Technologies

**DOI:** 10.1038/s41598-019-55322-4

**Published:** 2019-12-19

**Authors:** Spyros Stathopoulos, Loukas Michalas, Ali Khiat, Alexantrou Serb, Themis Prodromakis

**Affiliations:** Electronic Materials & Devices Research Group Zepler Institute for Photonics and Nanoelectronics University of Southampton, SO17 1BJ Southampton, UK

**Keywords:** Characterization and analytical techniques, Electrical and electronic engineering

## Abstract

The emergence of memristor technologies brings new prospects for modern electronics via enabling novel in-memory computing solutions and energy-efficient and scalable reconfigurable hardware implementations. Several competing memristor technologies have been presented with each bearing distinct performance metrics across multi-bit memory capacity, low-power operation, endurance, retention and stability. Application needs however are constantly driving the push towards higher performance, which necessitates the introduction of a standard benchmarking procedure for fair evaluation across distinct key metrics. Here we present an electrical characterisation methodology that amalgamates several testing protocols in an appropriate sequence adapted for memristors benchmarking needs, in a technology-agnostic manner. Our approach is designed to extract information on all aspects of device behaviour, ranging from deciphering underlying physical mechanisms to assessing different aspects of electrical performance and even generating data-driven device-specific models. Importantly, it relies solely on standard electrical characterisation instrumentation that is accessible in most electronics laboratories and can thus serve as an independent tool for understanding and designing new memristive device technologies.

## Introduction

Emerging memory-resistive devices, also known as memristors^1^, have exhibited an unmatched potential for a broad range of applications ranging from non-volatile memories^2^ to neuromorphic computing^3,4^ and reconfigurable circuits^5,6^. As the scope of these resistive memories expands, there is a growing interest in identifying all appropriate techniques for evaluating the different attributes of electrical performance^7^ and the physical aspects^8^ of Resistive Random Access Memory (RRAM) devices. While these techniques do offer valuable insights into the operation and underpinning physical aspects of devices they are limited to individual performance metrics. In order to establish a workflow to evaluate devices in a consistent manner that can be transferred across different laboratories a more *unified testing framework* is needed.

Our methodology presents a characterisation suite that allows to fully evaluate RRAM devices in a consistent and repeatable manner. Due to its all-electrical nature, it does not require using expensive equipment and its modular structure allows accessing insights on the underlying mechanisms without resourcing to complex and highly specialised equipment. Having multiple steps for testing a device in sequence empowers the user to cross-validate experimental observations as they occur through the complementarity of the individual modules. We endeavour not only to cover benchmarking performance aspects of the device but also capture signatures related to the underpinning switching mechanism, providing useful insights on device operation without the need for bespoke and expensive physicochemical validation tools. Our overarching aim is for this methodology to serve as an *independent tool* for pushing the development frontiers of novel memristive device technologies and their translation into emerging applications. Having a standardised methodology will also help with validating published data through repeating, without any ambiguity, memristors’ testing procedures.

**Figure 1:**
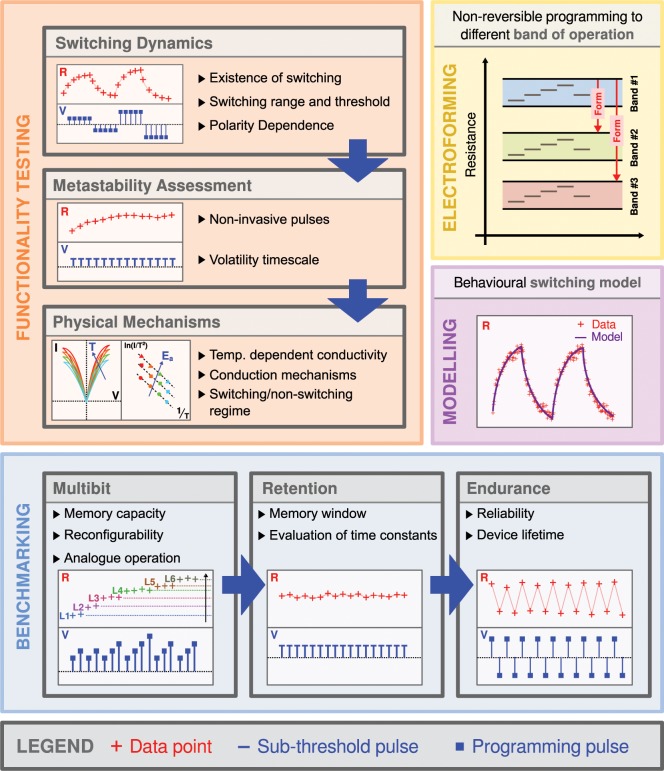
An overview of the proposed characterisation procedure introduced in this paper. Testing is split to four modules depending on their particular scope. *Functionality* testing establishes the switching capacity of the memristive cell as well as dominant conduction mechanisms and *Benchmarking* evaluates the actual performance of the device under test. Although Functionality and Benchmarking are to be understood as a sequence the operating range of the device can be tuned using the *Electroforming* procedure. *Modelling* is finally used to extract a behavioural model out of fully characterised devices.

The characterisation protocol, the overview of which can be seen in Fig. 1, consists of a series of consecutive modules each geared towards a specific performance target. Initially we deal with the *functionality* of the device itself, if any. In effect we query the capacity of any two-terminal device to act as a tuneable resistive element and determine its switching threshold and polarity dependency, forming a module we herein call *Switching Dynamics*. Next, we evaluate the stability of the Device Under Test (DUT) in its given resistive state thus evaluating the existence of volatile (metastable) dynamics. This is accomplished through a series of pulse stimuli, with voltage amplitudes below the switching threshold (sub-threshold) determined from the previous step. Our functionality testing is concluded with temperature dependent voltage cycling that can provide insights into the conduction mechanisms governing the switching in the DUT. This can be performed by considering the switching (supra-threshold) and the non-switching (sub-threshold) regimes of operation^9^.

Once a tuneable resistive functionality has been established, a series of benchmarking routines can be used to examine the actual performance metrics of the DUT. Given that a key feature of such elements is their history-dependence, we first evaluate the DUT characteristics with protocols that are the least possible to lead to an irreversible change in the device and then progressively increasing the applied stimuli. Initially, we employ a bespoke programming protocol to determine the ultimate memory capacity of the device in terms of number of non-volatile resistive states as a means to determine the potential of the device to operate in an analogue fashion, a key aspect of reconfigurable electronics. After the resistive states of the device have been identified, several retention steps across a series of these states can inform us of their stability as well as the DUT’s ability to retain the observed memory window that defines the dynamic range of switching. Moreover, another key metric is the number of cycles that a DUT can undergo before failing. Endurance testing can be performed in a bespoke manner, based on the intended use of operation, for example either between consecutive memory states or the extremes defining the DUT OFF/ON ratio.

We note that all of the above are dependent on the DUT preconditioning, often described as *electroforming*, a process that allows setting devices in distinct operating resistive “bands”. As electroforming fundamentally affects the physical characteristics of the DUT, both on a structural and interfacial level, this evaluation process should be repeated post-electroforming, or directly (without electroforming) for technologies that are electroforming-free.

Finally, to properly integrate a device into a circuit design workflow it is also important to have accurate behaviour models. The proposed memristor testing methodology thus culminates in the production of a phenomenological model that is driven by data and can closely match the response of the DUT for specific stimulus within the operating range that has been established throughout testing. Overall, the introduced methodology offers a holistic, yet versatile, characterisation routine that: (*i)* incorporates traditional techniques in standard use, (*ii)* introduces advanced techniques to capture finer effects and (*iii)* refines specialist techniques oriented towards understanding the underlying physical mechanisms.

## Functionality Testing

### Switching dynamics

**Figure 2:**
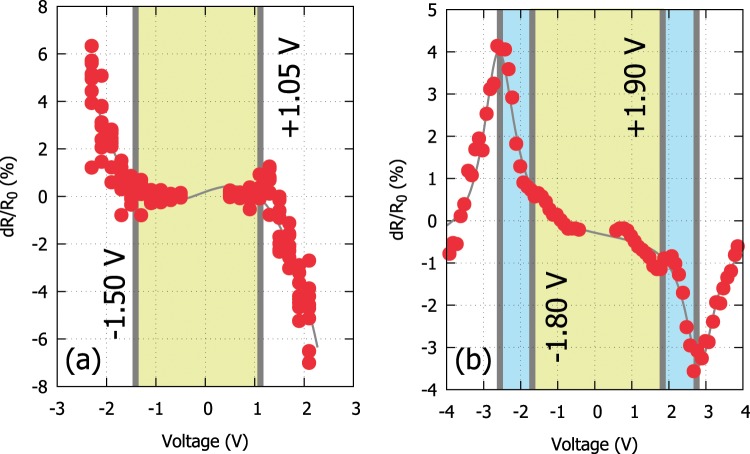
Switching dynamics. Relative resistance change in response to a given programming voltage in relation to its initial resistance (*R*_0_). Typical response of a Pt/TiO_2_/Pt device exhibiting bipolar behaviour (left) and a Au/TiO_2_/Pt exhibiting a hybrid unipolar/bipolar behaviour (right). The switching thresholds for an 1% change are highlighted. In the case of the second device the areas of unipolar operation are additionally highlighted. Programming pulse width is fixed at 1 μs.

First step towards characterising a new DUT should be the determination of the actual switching capability. This can be done by assessing its switching dynamics by employing a two-stage characterisation algorithm^10^. Initially pulses of alternating polarity are applied to the DUT for determining the direction of change in the resistance of the device for a provided stimulus polarity. Then, voltage ramps are used to determine the actual change in the resistance. As a typical example of this routine can be seen in we can observe in Fig. 2 the relative resistive response of two different devices (Pt/TiO_2_/Pt and Pt/TiO_2_/Au at the ~30 kΩ and ~5 kΩ operating range) after the application a series of 100 μs wide pulses of gradually increasing amplitude. From these experimental data one can establish the switching regime for the two DUTs. In these examples, the first shows a clear bipolar response to pulses of different polarity, which is the typical behaviour expected for a bistable memory device. However, the second device exhibits a hybrid bipolar/unipolar behaviour where the applied bias can result to both an increase and a decrease of the device resistance depending on the stimulus amplitude when the applied bias surpasses a threshold and the behaviour of the device changes to unipolar. Although in the first case the operating boundaries of the device are clear, in the second case this analysis allows us to establish an operating voltage range where the device remains strictly within the bipolar switching regime, depending to one’s application needs. This tool is essential for identifying appropriate biases for any DUT and for establishing its switching threshold for a pre-defined pulse width.

It is important to mention that capacitance (either of the measuring system or the device itself) may affect the quality of the applied pulses leading to bandwidth limitations. This could have a detrimental effect on the outcome. In our approach we do not aim to differentiate between devices with different high-frequency characteristics but eliminate such differences by necessitating pulses with fast rise and fall times (or respectively longer pulse widths) in order to impose a quasi-DC operating regime.

### Switching metastability evaluation

Once a DUT functionality is established, we proceed with assessing its *metastability* or *volatile* characteristics, i.e. the attribute of several memristive technologies to revert back to their initial resistive state. This is accomplished through the use of non-switching pulses that allow reading the DUT resistive state without affecting it directly, i.e. without switching the DUT any further. Such variations in the resistance are correlated with the inherent instability of the device itself, for example due to re-oxidation^11^ and/or other mechanisms^12^, based on the device structure. For DUTs that exhibit this kind of volatility, it is crucial to determine the timescale of said variations in order to accommodate for that in experiment planning and design of applications.

### Temperature dependent I-V characterisation

**Figure 3:**
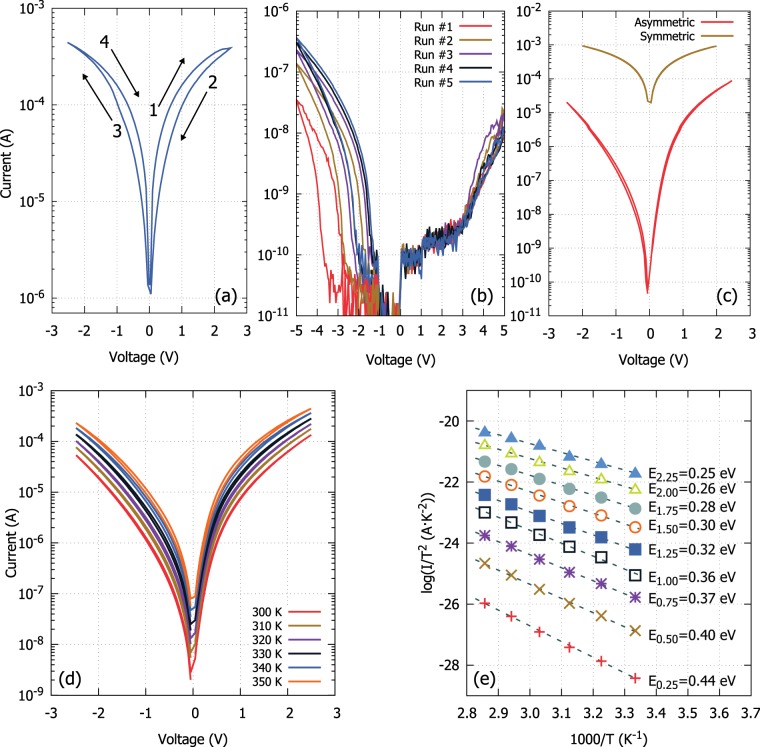
Physical mechanisms through I-V characteristics. (**a**) Regular gradual switching I-V of a bipolar device (Pt/TiO_2_/Pt – electroformed device). (**b**) Hysteresis loop and drift of the I–V arise by the multiple acquisition iterations. This behaviour denotes strong ionic character in the core metal oxide thin film (Pt/TiO_2_/Pt – pristine device). (**c**) Typical asymmetric (red) and symmetric (yellow) I–Vs obtained on a Pt (TE)/TiO_2_/Au (BE) and on a Al (TE)/TiO_2_/Au (BE) respectively. This is an initial indication of interface and material controlled transport. (**d**) Temperature analysis for pristine Metal-Oxide-Metal stacks depicting evolution of I–V characteristics and (**e**) temperature dependent signature plots deriving from them following the analysis presented in^16^. This analysis allows for identifying the dominant transport mechanism. This particular case corresponds to Schottky emission^13,16^.

Perhaps the most celebrated testing procedure of memristive technologies is capturing their current-voltage characteristics (I–Vs). Recording of the I–V DUT signature provides: (*i)* a *straightforward testing* for the device operation (*ii)* indications for any type (volatile or stable) of *switching capability* and (*iii)* a deeper insight on the *device physics*, i.e. the mechanism underneath the electrical response. Recording of the DUT I–V characteristic signature provides an initial indication of its resistive switching character, i.e. bipolar or unipolar, whilst it leads in qualitatively identifying the SET/RESET behaviour. As one of the prime characteristics of memristive devices is their history dependence, a bias ramp that constitutes a succession of pulse stimuli with a step-increased amplitude will in principle have a cumulative effect and thus any observable switching at a given stimulus amplitude cannot be expected to be repeatable with a single pulse of same amplitude. The extracted information can also be used on subsequent characterisation steps, such as the endurance and/or multistate memory evaluation routines, described in more detail in the following sections. The identified operating switching characteristics (Fig. 3a) can also be used to complement and corroborate those attributes identified through the *switching dynamics* evaluation routine.

Besides providing valuable switching performance characteristics, the I–V evaluation is also capable of revealing the nature of the underlying switching mechanism. An unstable/non-reproducible I–V (Fig. 3b), could be associated with metastability induced by movable ions, associated to the oxygen vacancies or even interstitials. Movable ions, however, can also elicit stable (non-volatile) transitions that result in stable I–Vs presenting notable hysteresis loops on a full acquisition cycle. The ions contribution to the DUT’s conduction could be direct or indirect, for example through modifying the charge close to the interface and in consequence the potential barrier, overall resulting in a resistance modulation. An asymmetric I-V response with respect to the bias polarity is an indication of transport determined by the interface barriers whilst a symmetric curve should be associated with core-material controlled transport (Fig. 3c).

The in-depth clarification of the transport properties requires assessing the temperature dependence of the DUT’s I–V. This is because the various conduction mechanisms obey both field- and temperature-dependencies^13^ and thus the recording of the I–V curves at different temperatures (Fig. 3d) allows the extraction of relevant characteristic signature plots. This could take the form of simple Arrhenius plots or more sophisticated versions when the dominant mechanism involves processes such as hopping, Frenkel-Poole emission^14^ or Schottky emission over an interface barrier^15,16^ (as depicted in Fig. 3e). Further assessment of such signature plots allows for quantitatively extracting parameters related to the DUT’s interface barrier height or the activation energy of the involved defects, in the cases of interface and material-controlled transport, respectively. It is worth mentioning to the case where clear indications of filamentary switching is present. In this case temperature analysis should be carefully applied as the local heating effect of the constricted conductive path may result in the actual localised temperature to be different than what is measured macroscopically. In this particular case more sophisticated techniques may be required to decouple localised from macroscopic heating^17^.

## Electroforming

RRAM technologies can be operated across a variety of resistive regimes and considering also the application needs, pristine devices might require an electroforming step for bringing these into a desirable resistive range. Electroforming was originally mentioned by Hickmott^18,19^ where he describes it as an *irreversible* change of the electrical properties of the material by applying a voltage greater than a minimum *forming* voltage. The electroforming process can represent either the formation of a conductive filament due to structure altering effects or oxygen deprivation^20^, the diffusion of metal into the metal oxide layer or the modifying the interfacial barrier between the electrodes and the metal oxide active layer^21,22^. At this point the core material and the interfaces of the resistive memory stack have been completely reformed with respect to their prior state. It is therefore essential to re-evaluate the functionality characteristics of the DUT. In addition, this allows for correlating the pre- and post-formed characteristics, enabling new opportunities for tuning the performance of the device by means of optimising the applied pulsing scheme.

**Figure 4:**
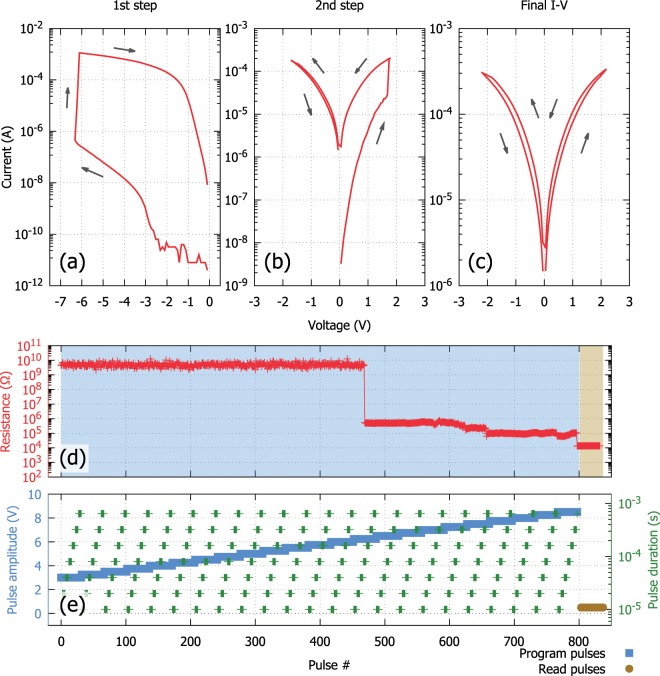
Electroforming schemes. (**a–c**) Two step electroforming using staircase I-V of a Pt/TiO_2_/Pt device. Apparent electroforming voltage from the GΩ range is about −6.5  V (left). The device is undergoing a further electroforming step bringing it down to the 30  kΩ range (middle) where a steady state is established (right). (**d,e**) Typical two step electroforming process using a pulse sequence. This device is formed with pulses of increasing amplitude and pulse width. Target resistance threshold has been set to 10  kΩ. The device initially drops to the MΩ range before attaining its final value. Highlighted in blue is the biasing region, followed by a series of READ pulses (highlighted in yellow). Readout pulse width during programming ranges from 20 ms in the GΩ range down to 1  ms in MΩ and below range.

Regardless of the actual forming mechanism, during this process, the resistance of the device is lowered to ranges that are typically relevant to applications. It is well-known^18,19^ that electroforming is not possible before a device-dependent voltage threshold is reached; the way to cross this threshold is, however, not immediately obvious. One way to form a device is through an I–V cycle, as shown in Fig. 4a. The DUT is biased with increasingly higher voltage steps until a significant change in the DUT’s resistance is observed. We note, that further steps might be required to bring the device into a non-volatile switching regime. As observed in Fig. 2b, the conductance of the DUT is still lower than the one achieved in the first step. In this case, a further forming cycle is required (shown in Fig. 4b) before reaching a final state (Fig. 4c). Further increasing the voltage amplitude leads to a partial dielectric breakdown^23^ of the active layer and *compliance* is an issue that must be dealt with during electroforming in order to prevent irreversible switching degeneration of the device. Two distinct forms of compliance can be identified: *current compliance* and *time compliance* (i.e. short controllable pulses). The key issue with the current compliance mode is that there is a distinct delay before reaching the current cut-off threshold with current overshoot, partly due to a delayed response of the compliance system as well as due to residual parasitic capacitance^24^. As such, *short sequential pulses* can be a better alternative for a controllable forming procedure. Instead of continuously biasing the device, sequential voltage pulses of continuously increasing duration and amplitude are applied to the device. This approach offers a less invasive procedure towards attaining a desired formed state. In the example shown in Fig. 4, a series of programming pulses from 3 to 10 V is applied to the device. Within each step the pulse duration is modulated from the low-μs up to the ms range.

In addition, Fig. 4 further illustrates the common pattern in the electroforming process where there is an initial increase in the electrical conductivity of the DUT, marking the onset of the electroforming mechanism. By continuing the application of the programming pulses the device is finally driven to its target resistive state. While after the first forming the device is able to be tuned to a multitude of resistive states the initial increase to the conductance of the device is irreversible as it is associated with morphological alterations of the active layer^25^. This behaviour is also evident in the electroforming procedure using staircase I-V curves, as depicted in Fig. 4a–c.

## Performance Metrics

### Memory capacity – multibit evaluation

All of the previous steps mostly focus on the evaluation of the switching behaviour of the device. Broaching into the area of applications of particular importance is the ability to identify the memory capacity of the device. Although the bistable aspect of the device operation is considered straightforward, driving a DUT at intermediate states within these two extreme boundaries, requires a more targeted characterisation approach. The need for multibit memory capacity in conventional RRAM cells is nowadays gaining more importance thanks to a series of emerging applications of memristor technologies in reconfigurable circuits and neuromorphic computing.

**Figure 5:**
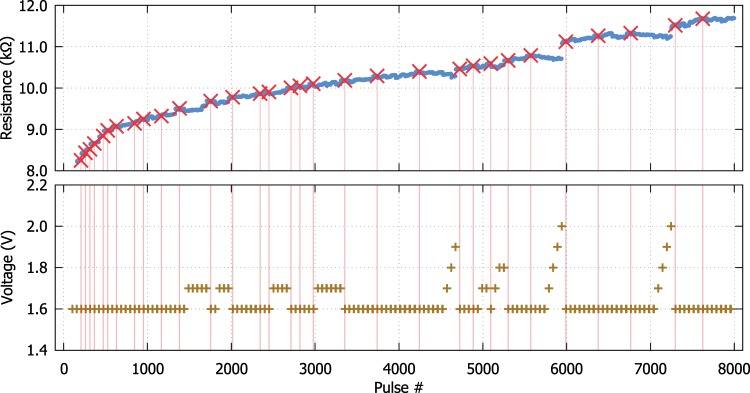
Multibit evaluation. Memory capacity of a Pt/TiO_2_/Pt RRAM cell. In the top figure stable resistive states have been marked with red crosses. The corresponding programming protocol is shown in the bottom figure. Read pulses are applied continuously throughout and are set to 0.2  V.

Along this line, a comprehensive characterisation routine can be used as described in a previous publication^26^. A succession of fixed pulse width pulse trains each containing an increasing number of programming pulses is applied to the DUT followed by a short retention test to assess the stability of the current resistance. If during this test the resistance of the DUT remains within a pre-defined tolerance band then a new state is registered. Otherwise, the amplitude of the bias is increased up to a specified limit and a new succession of pulse trains in applied. In Fig. 5 one can observe a typical multi-level memory characterisation output for a Pt/TiO_2_/Pt RRAM cell. In this case, the read voltage is kept at 0.2 V and the characterisation protocol is applied for up to 10 pulses of 1 μs wide programming pulses ranging in amplitude from 1.6 V to 2.1 V with a confidence interval of 2σ. In this example, 5 bits of information (31 states) can be extracted from the DUT within just 4 kΩ of resistance span (8 to 12 kΩ). Depending on the target application, the confidence intervals can be accordingly adjusted to allow for either a high number of states or a larger interval in terms of resistance between the states.

### Base performance evaluation (Endurance and Retention)

**Figure 6:**
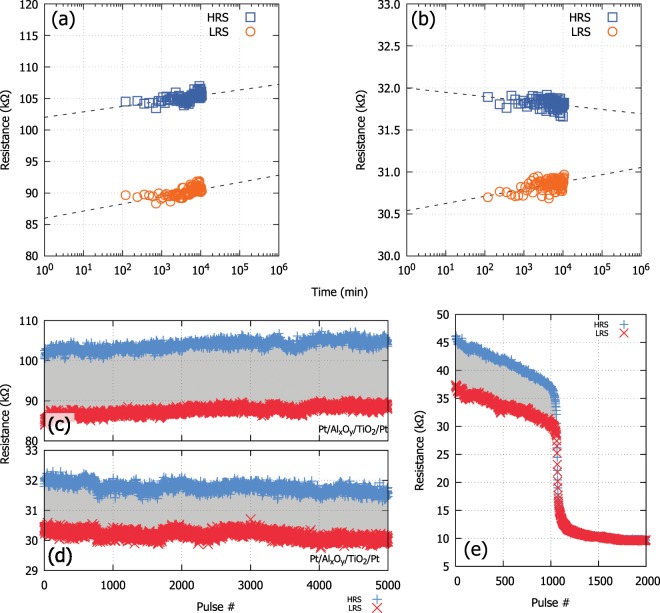
Performance metrics. (**a,b**) Retention measurements (room temperature) for the two different resistive states defined in Fig. 4a,b. Read voltage set at 0.2  V. By extrapolating the retention sampling data the device on the left retains its memory window despite the drift in resistance, whereas the device on the right has seen a deterioration to its window for the same period. (**c–e**) Endurance characterisation of two different devices. On the left (subfigures c and d) a Pt (TE)/Al_x_O_y_/TiO_2_/Pt (BE) is subjected to 5000 pulses of 2  V pulses of alternating polarity for two different resistive ranges. In both cases the resistive window is maintained throughout. On the right (**e**) a Pt (TE)/TiO_2_/Pt (BE) device exhibits a deteriorating memory window which is completely eliminated after 1000 pulses thus quickly failing the endurance test. Resistive lifetimes and windows are technology specific.

Although devices can alternate between neighbouring or extreme states the retention of such memory windows is not certain. To ascertain the stability of this window that defines the dymanic range of switching for a DUT, long retention tests are required where the DUT is programmed to different resistive levels within the operating range of the device and read continuously for prolonged periods of time. A typical output of such experiment can be seen in Fig. 6a. In this case, the DUT is continuously read for a period of up to three hours for two different states. The data are extrapolated for a period of 10 years (~10^6^ minutes) so that the stability of the memory window defined by the resistive states can be established over that period. A different example is shown in Fig. 6b where the switching window is considerably degraded.

In order to determine how any DUT behaves under repeated stress an endurance test is required. For a typical bipolar DUT, a series of alternating polarity pulses is applied to the device switching it between neighbouring resistive states such as those defined from the retention test previously applied. A typical output can be seen in Fig. 6c,d where 2 V, 100 μs wide pulses of alternating polarity are applied to a Pt (TE)/Al_x_O_y_/TiO_2_/Pt (BE) device for two different resistive ranges. The ideal technology would retain its memory window for as long as possible, subject to biasing, while a failing device (as the case depicted in Fig. 6e) will have its memory window quickly deteriorating to a point that it is completely eliminated.

Base performance evaluation is a valuable tool as it can be used for the derivation of further performance metrics as for instance power dissipation an upper bound of which can be estimated by time-integrating the product of measured current and applied voltage.

## From devices to Applications – Phenomenological Modelling

In order to properly integrate memristive technologies into an integrated circuit workflow it is essential to have realistic, accurate and computationally efficient behavioural models extracted from readily available data. The proposed methodology aids in that direction by providing a standardised approach. Our methodology makes it is possible to provide enough data to instantiate an phenomenological model that describes this DUT where the rate of change of the resistance is modelled a function of the resistance itself (*R*) and the applied bias (*v*). Such a model is described in a previous publication^27^.

$$\frac{dR}{dt}=s(v)\times f(R,v)$$

where *s*(*v*) is the switching sensitivity and *f*(*R*, *v*) the window function.

$$s(v)=\{\begin{array}{c}{A}_{p}(-1+\exp (\frac{|v|}{{t}_{p}})),\,v > 0\\ {A}_{n}(-1+\exp (\frac{|v|}{{t}_{n}})),\,v < 0\end{array}\,{\rm{and}}\,f(R,v)\{\begin{array}{c}{({a}_{0,p}+{a}_{1,p}v-R)}^{2},v > 0\\ {(R-{a}_{0,n}+{a}_{1,n}v)}^{2},v < 0\end{array}$$

**Figure 7:**
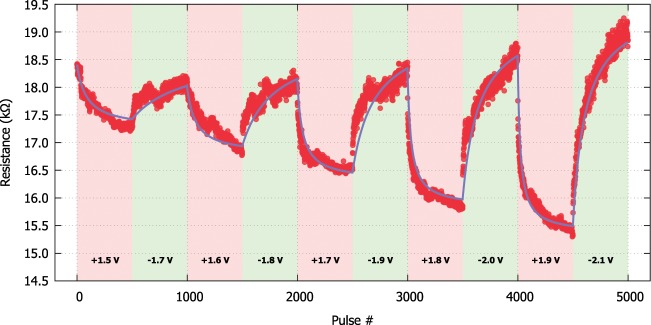
Behavioural model fitting. The analytical model (solid blue line) extracted from the resistive response of a Pt/TiO_2_/Pt RRAM cell (red dots) using 500 pulse batches of alternating polarities. The amplitude of each of the pulse trains applied is indicated on the bottom of the graph. The initial resistance of the device is 18.3  kΩ.

The rest of the parameters are free fitting variables for the positive and negative branch of the bias. Data can be obtained as part of this characterisation procedure by applying a fixed number of programming pulses, while alternating the polarities and fitting the above equations in a least square fashion. An example is showcased in Fig. 7 where a bipolar Pt/TiO_2_/Pt RRAM cell is biased with alternating programming pulses of increasing amplitude ranging in amplitude from 1.5 V to 1.9 V and −1.7 V to −2.1 V. The fitting parameters for this DUT are summarised in Table 1.

**Table 1:** Fitting parameters for the phenomenological model used in this example.

Positive pulses	Negative pulses	Units
*A*_*p*_ = −0.14	*A*_*n*_ = 0.02	*Ω*·*s*^−1^
*t*_*p*_ = 2.74	*t*_*n*_ = 3.03	*V*
*a*_0*p*_ = 24.4×10^3^	*a*_0*n*_ = 14.7×10^3^	*Ω*
*a*_1*p*_ = −4.75×10^3^	*a*_1*n*_ = −2.33×10^3^	*Ω*·V^−1^

In summary, in this paper we presented a complete testing methodology that can be used for benchmarking the performance of emerging memristive device technologies. The methodology covers all technological facets required to integrate a new resistive memory technology on any workflow, starting from fundamental physical aspects to performance characteristics memory capacity and behavioural modelling.

## Methods

### Device fabrication

Devices used in this paper have been fabricated on 6-inch oxidised silicon wafers (200 nm of thermal SiO_2_. Bottom electrodes were fabricated using photolithography and electron beam evaporation of titanium and platinum ( 20 nm) or gold (10 nm) followed by lift-off process in N-Methyl-2-pyrrolidone (NMP). Then 25 nm of TiO_2_ were deposited using magnetron sputtering. For the bi-layer devices an additional 4 nm of Al_x_O_y_ was deposited using the same process. Top electrodes were again fabricated with photolithography, electron beam evaporation or sputtering of platinum (10–20 nm) and lift-off in NMP. Devices with active areas of 20 × 20 μm² and 30 × 30 μm² were used for the purposes of this paper. Discussion on the morphology of the films used as well as optical images of the devices can be found in previous publication^16^.

### Electrical characterisation

All characterisation of the devices has been done with our characterisation platform ArC ONE^28^, although the methodology itself is instrumentation independent. Read pulses are set to up to 50 ms in duration (depending on actual resistive state) and 0.2 V in amplitude. Nominal line resistance for the devices is estimated to be 50–250 Ω depending on electrode material and length. Depending on the stack a pulsing-based electroforming routing has been used with consecutive 1 μs to 1 ms pulses ranging from ±6 to ±12 V in amplitude using an 1 kΩ resistor for current limiting. Endurance testing was performed with single alternating polarity pulses as per the article and retention measurements have been performed over a period of 3 hrs using the readout scheme described above. For the I–V curves 2–50 ms pulses were applied at the specific amplitude while measuring the resistance. The inter-pulse interval between consecutive pulses is either 0 ms (staircase mode) or 1 ms (pulsed mode). Multi-bit capability of the devices is assessed using a custom three-phase algorithm outlined in a previous publication^26^. For experiments where temperature control was required we used an ESPEC ETC-200L temperature controller with applicable temperature range of 0–200 °C. Thermal chuck was set to a specific temperature and the wafer was allowed to thermally stabilise so as to obtain the I-V curve at thermal equilibrium.

## Data Availability

The data that accompany this study are available from the University of Southampton institutional repository at 10.5258/SOTON/D1153.
